# Unraveling the Complex Trait of Harvest Index with Association Mapping in Rice (*Oryza sativa* L.)

**DOI:** 10.1371/journal.pone.0029350

**Published:** 2012-01-23

**Authors:** Xiaobai Li, Wengui Yan, Hesham Agrama, Limeng Jia, Aaron Jackson, Karen Moldenhauer, Kathleen Yeater, Anna McClung, Dianxing Wu

**Affiliations:** 1 State Key Lab of Rice Biology, International Atomic Energy Agency Collaborating Center, Zhejiang University, Hangzhou, People's Republic of China; 2 Agricultural Research Service, United States Department of Agriculture, Dale Bumpers National Rice Research Center, Stuttgart, Arkansas, United States of America; 3 Institue of Horticulture, Zhejiang Academy of Agricultural Sciences, Hangzhou, People's Republic of China; 4 University of Arkansas, Rice Research and Extension Center, Stuttgart, Arkansas, United States of America; 5 Agricultural Research Service, United States Department of Agriculture, Southern Plains Area, College Station, Texas, United States of America; United States Department of Agriculture, Agricultural Research Service, United States of America

## Abstract

Harvest index is a measure of success in partitioning assimilated photosynthate. An improvement of harvest index means an increase in the economic portion of the plant. Our objective was to identify genetic markers associated with harvest index traits using 203 *O. sativa* accessions. The phenotyping for 14 traits was conducted in both temperate (Arkansas) and subtropical (Texas) climates and the genotyping used 154 SSRs and an *indel* marker. Heading, plant height and weight, and panicle length had negative correlations, while seed set and grain weight/panicle had positive correlations with harvest index across both locations. Subsequent genetic diversity and population structure analyses identified five groups in this collection, which corresponded to their geographic origins. Model comparisons revealed that different dimensions of principal components analysis (PCA) affected harvest index traits for mapping accuracy, and kinship did not help. In total, 36 markers in Arkansas and 28 markers in Texas were identified to be significantly associated with harvest index traits. Seven and two markers were consistently associated with two or more harvest index correlated traits in Arkansas and Texas, respectively. Additionally, four markers were constitutively identified at both locations, while 32 and 24 markers were identified specifically in Arkansas and Texas, respectively. Allelic analysis of four constitutive markers demonstrated that allele 253 bp of RM431 had significantly greater effect on decreasing plant height, and 390 bp of RM24011 had the greatest effect on decreasing panicle length across both locations. Many of these identified markers are located either nearby or flanking the regions where the QTLs for harvest index have been reported. Thus, the results from this association mapping study complement and enrich the information from linkage-based QTL studies and will be the basis for improving harvest index directly and indirectly in rice.

## Introduction

In food production, optimizing grain yield, reducing production costs, and minimizing risks to the environment have been the primary objectives since the beginning of the twentieth century [1]. Food crops grow by developing a vegetative canopy that transpires water and carries out photosynthesis, and a root system that takes up water and nutrition, which leads to the production of biomass. Following the reproductive stage, a portion of the plant biomass is partitioned to various yield components and determines harvest index [2] Harvest index is the ratio of grain yield to total biomass and is considered as a measure of biological success in partitioning assimilated photosynthate to the harvestable product [3], [4], [5]. In cereal crops, dramatic improvements in harvest index have made commercial cultivars greatly different from their wild ancestors [6]. Rice (*Oryza sativa* L.) is one of the most important staple foods [7]. It can be highly productive if high harvest index genotypes are grown with optimal management practices [2]. Harvest index of rice is the result of various integrated processes with an involvement of the number of panicles per unit area, the number of spikelets per panicle, the percentage of fully ripened grains, and the weight of 1,000 mature kernels [8]. Marri et al. [9] found that harvest index was negatively correlated with plant height, but positively correlated with grain number/panicle, grain number/plant, percentage spikelet fertility, test grain weight and yield/plant in rice. Sabouri et al. [10] verified the negative correlation of harvest index with plant height and positive correlation with spikelet number and grain weight per panicle, and reported the impact of some flag leaf characteristics on harvest index in rice. In maize, harvest index is negatively correlated with plant height, but positively correlated with grain yield both phenotypically and genotypically [11]. In sorghum, harvest index is negatively correlated with forage yield [12], but positively correlated with growth rate and grain filling rate [13]. Usually, the correlated traits are interrelated, so that increases in one component may lead to decreases or increases in others. Therefore, scientists aim to identify genes/QTLs that directly improve a target trait without negatively affecting others, or improve the target trait indirectly through the improvement of its associated characteristics.

Crop harvest index is also highly influenced by environmental factors [14], such as soil condition [15], [16] and temperature [17], [18]. However, genetic control of harvest index plays important role in crop production. Large variation was observed for harvest index in rice: about 0.25 among wild species, 0.30 among tall cultivars and more than 0.40 for semi-dwarf cultivars [19]. The intrinsic regulation of harvest index is controlled by many genes. A few reports in the literature have examined QTLs in rice associated with harvest index. Mao et al. [20] reported four main-effect QTLs for harvest index on chromosome (Chr) 1, 4, 8 and 11 and other epistatic interaction between two QTLs respectively on Chr 1 and Chr 5. Sabouri et al. [10] identified three QTLs mapped on Chr 2, 3 and 5, and two QTLs close to each other on Chr 4. Lanceras et al. [21] described harvest index QTLs on Chr 1 and 3. However, a recurring complication of the QTL data showed that different parental combinations and/or experiments conducted in different environments often result in partly or wholly non-overlapping sets of QTLs [22]. Therefore, it is necessary to explore constitutive QTLs across different environments and adaptive QTLs specifically for a given environment [23].

Classical QTL mapping reveals only a portion of the genetic control of a trait because there are only two alleles that can differ at any locus between the two parental lines. More comprehensive analyses of genetic architecture require consideration of a larger sample of the genetic variation in the species. One approach is association mapping, which maps the QTLs either among extant breeding lines with known pedigree relationships or in a diverse germplasm collection. Given pedigree and marker information, the probability for different lines in complex populations to share identity by descent QTLs can be defined, permitting estimation of the effects of each QTL [24]. Association mapping provides an alternate route into identifying the QTLs that have effects across a broader spectrum of germplasm, if false-positives caused by population structure can be minimized [25]. Whole-genome association scans are expected to be effective when linkage disequilibrium (LD) and marker density are sufficiently high, so that the random markers could have a greater chance of being in disequilibrium with QTLs across diverse genetic materials [26]. Huang et al. [27] successfully performed genome-wide association study (GWAS) in a rice landrace collection of China for 14 agronomic traits and identified a substantial number of loci at close to gene resolution. Many other studies have minimized the large-scale population structure effects by analyzing associations separately for each heterotic group, and controlled the finer-scale population structure by explicitly incorporating pedigree relationships between lines in the analysis [25], [26], [28], [29], [30], [31], [32].

Recently, the USDA rice mini-core (URMC) subset was developed and serves as a genetically diversified panel for mining genes of interest to various users [33]. The URMC was derived from 1,794 accessions in the USDA rice core collection using PowerCore software based on 26 phenotypic traits and 70 molecular markers [34]. The core collection represents over 18,000 accessions in the USDA global genebank of rice [35]. The URMC contains 217 accessions originating from 76 countries and covering 14 geographic regions worldwide plus some of unknown origin. The URMC has a great genetic diversity and well represents the five sub-populations found in *O. sativa* [33]. As a result, it is an ideal population for exploring QTLs responsible for harvest index traits with the powerful approach of association mapping.

We genotyped 203 O. *sativa* URMC accessions with 155 molecular markers and phenotyped 14 traits contributable to harvest index in both temperate (Stuttgart, Arkansas) and subtropical (Beaumont, Texas) locations. Our objectives were to identify the traits significantly correlated with harvest index *per se* and the markers significantly associated with component traits of harvest index. To control spurious associations, i.e., Type I error, we analyzed the genetic structure and familial relatedness in the collection. Different mapping models were tested for best fit of each trait. The chosen model was used to map markers associated with harvest index and associated traits phenotyped in two environments.

## Results

### Markers profile

The set of 154 SSRs and an *indel* with genome-wide distribution detected a total of 1993 alleles among 203 *O. sativa* accessions. The average number of alleles per locus was 12.86 ranging from 2 for RM338 to 57 for con673. Polymorphic Information Content (PIC) varied from 0.25 for AP5625-1 to 0.97 for con673 among the 155 markers with an average of 0.71. Nei's (1983) [36] genetic distances ranged from 0.0181 to 0.9667 with an average 0.7464 among each pair of 203 accessions in the URMC.

### Population structure and geographic origin

**Figure 1:**
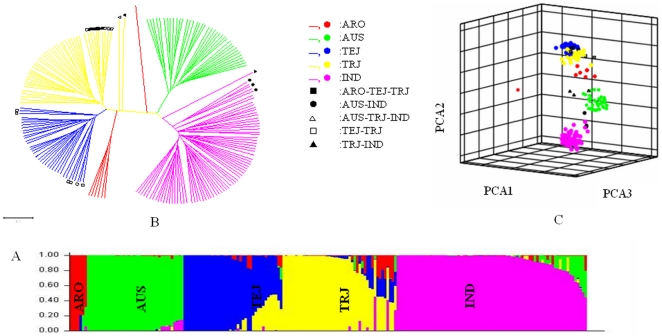
Structure analysis of USDA rice mini-core collection using A: *STRUCTURE*, B: Unrooted UPMGA and C: PCA. ARO: *aromatic* in red; AUS: *aus* in green; IND: *Indica* in purple; TRJ: *Tropical japonica* in yellow; TEJ: *Temperate japonica* in blue; ARO-TEJ-TRJ: admixture of ARO with TEJ and TRJ; AUS-IND: admixture of AUS with IND; AUS-TRJ-IND: admixture of AUS with TRJ and IND; TEJ-TRJ: admixture of TRJ with TEJ; TRJ-IND: admixture of TRJ with IND.

Using *STRUCTURE* software with multi-loci genotype data, a five-group model was identified to sufficiently explain genetic structure among 203 accessions. Ancestry of each of these accessions was inferred for assignment into a genetic group (Figure 1A). A dendrogram tree created with *PowerMarker* had five main branches for the 203 accessions as well (Figure 1B). The principal components analysis (PCA) also displayed the pattern of genetic structure with five groups. The first three components of PCA for 45.07% of total variation were used to visualize the five groups derived from ancestry analyses (Figure 1C).

**Figure 2:**
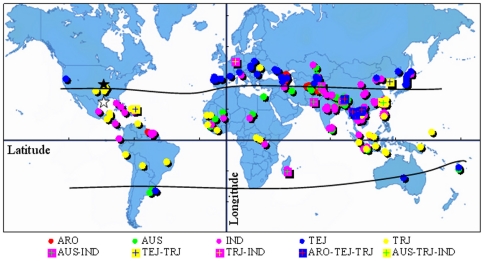
Geographic distribution of 203 accessions based on their latitude and longitude. ARO: *Aromatic*; AUS: *aus*; IND: *Indica*; TRJ: *Tropical japonica*; TEJ: *Temperate japonica*; ARO-TEJ-TRJ: admixture of ARO with TEJ and TRJ; AUS-IND: admixture of AUS with IND; AUS-TRJ-IND: admixture of AUS with TRJ and IND; TEJ-TRJ: admixture of TEJ with TRJ and TRJ-IND: admixture of TRJ with IND. ★: Stuttgart AR, ⋆: Beaumont TX.

The resultant five groups of *O. sativa* categorized by the Q value (ancestry index) belong to *indica* (IND), *temperate japonica* (TEJ), *tropical japonica* (TRJ), *aus* (AUS) and *aromatic* (ARO) (Figure 1A), based on reference cultivars reported previously by Garris et al. [37], Agrama and Eizenga [38] and Agrama et al. [34]. Each accession with ancestry information was plotted on a world map using its latitude and longitude of geographic origin (Figure 2). TEJ accessions were mainly distributed between latitudes 30 and 50 degrees north and south of the equator (i.e. temperate zone) while the other four groups scattered between latitude N 30 and S 30 degrees (i.e. tropical and subtropical zone).

### Morphological analysis

**Figure 3:**
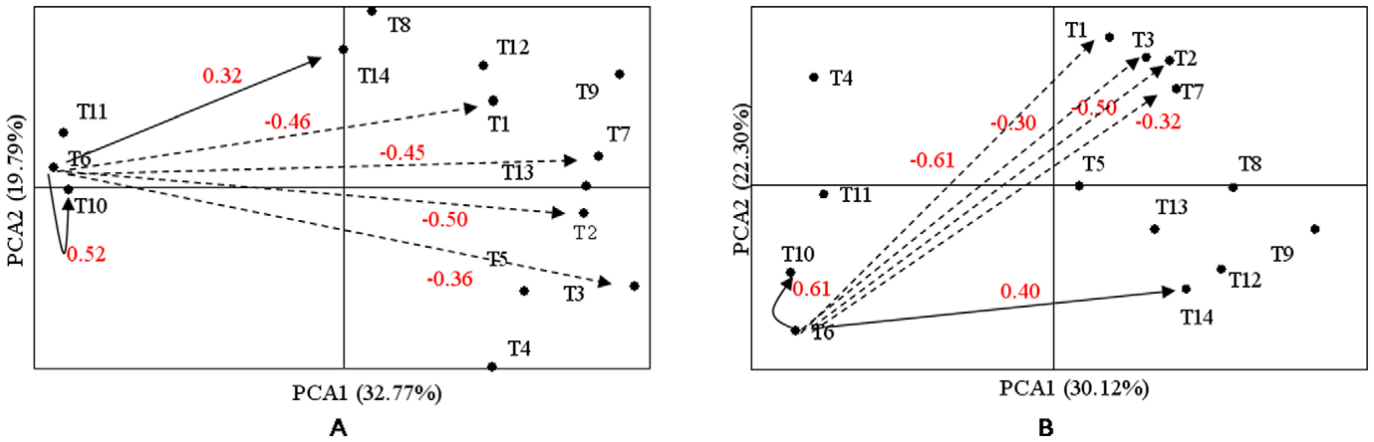
Relationship map constructed by PCA for 14 traits at A: Stuttgart, AR and B: Beaumont, TX. The distance between traits is inversely proportional to the size of the correlation coefficients. *Solid* and *dashed lines* indicate positive and negative correlations, respectively. Trait names are T1:Heading; T2:Plant height; T3:Plant weight; T4:Tillers; T5:Grain yield; T6:Harvest index; T7:Panicle length; T8:Panicle branches; T9:Kernels/panicle; T10:Seed set; T11:1000 Seed weight; T12:Kernels/cm panicle; T13:Kernels/branch panicle; T14: weight/panicle. The variation explained by the principal components is showed in the brackets.

Statistical analysis using a mixed model demonstrated that the differences due to genotypes and genotype×location interactions were highly significant at the 0.001 level of probability for all of the 14 traits (Table 1). The differences due to location were also significant for 12 traits except for panicle branches and seed set. Heritability was very high for all of these 14 traits. Heading had the highest heritability which was close to 100%. Although seed set had the lowest heritability, it was still above 70%. Heritability ranged from 77 to 97% among the other 12 traits. Harvest index had a heritability of 83% at Stuttgart and 90% at Beaumont. Correlation coefficients for each pair of the 14 traits were calculated using Spearman rank for each location and presented in Table S1A and S1B, respectively. To visualize the complex relationship among the 14 traits, PCA was used to construct plots with the first two axes accounting for more than 50% phenotypic variation (Figure 3A, B). At Stuttgart, 47 out of 91 correlations among the 14 traits were significant (<0.0001) (Table S1A, Figure 3A), and 40 correlations were significant at Beaumont (Table S1B, Figure 3B). Thirty four correlations were uniformly significant across two locations and their correlation directions (positive or negative) were also same across two locations (Table S1A, S1B).

**Table 1:** Statistical analysis of 14 traits generated at Stuttgart, Arkansas and Beaumont, Texas in 2009 in the USDA rice mini-core collection.

Trait	Location	Mean ± SD	Range	Heritability (%)	Genotype		Location		Genotype*Location	
					F value	Pr>F	F value	Pr>F	F value	Pr>F
Heading (days)	Stuttgart	99.33±21.31	34.00∼181.67	98.08	341.53	0.000000	2634.77	0.000001	12.45	0.000000
	Beaumont	87.55±22.63	38.00∼182.00	98.64						
Plant height (cm)	Stuttgart	109.73±20.20	61.08∼153.92	97.11	127.48	0.000000	1676.50	0.000002	45.63	0.000000
	Beaumont	124.74±22.45	67.00∼178.78	95.73						
Plant weight (g)	Stuttgart	168.71±79.88	27.83∼548.42	86.33	30.87	0.000000	122.48	0.000376	9.94	0.000000
	Beaumont	219.02±87.70	35.93∼558.02	86.83						
Tillers	Stuttgart	23.95±11.20	6.42∼67.75	86.53	35.27	0.000000	818.76	0.000009	7.10	0.000000
	Beaumont	41.13±15.83	13.00∼85.89	87.16						
Grain yield (g)	Stuttgart	60.02±25.51	8.54∼127.27	87.05	29.06	0.000000	98.37	0.000568	8.33	0.000000
	Beaumont	76.67±30.05	5.64∼165.97	84.03						
Harvest index (%)	Stuttgart	30.44±7.02	3.40∼45.06	82.75	35.79	0.000000	2174.76	0.000000	6.10	0.000000
	Beaumont	38.98±10.51	6.25∼60.02	89.98						
Panicle length (cm)	Stuttgart	26.66±3.81	14.21∼37.19	89.86	46.56	0.000000	293.26	0.000060	3.68	0.000000
	Beaumont	24.75±3.44	16.84∼38.40	88.34						
Panicle branches	Stuttgart	10.97±2.15	5.44∼17.78	85.65	29.97	0.000000	31.18	0.004559	2.40	0.000000
	Beaumont	10.64±2.06	5.56∼16.33	81.68						
Kernels/panicle	Stuttgart	194.97±57.49	68.56∼399.00	86.48	29.94	0.000000	367.90	0.000041	4.45	0.000000
	Beaumont	155.77±45.46	50.00∼318.33	86.92						
Seed set (%)	Stuttgart	78.15±15.23	25.48∼96.97	78.39	15.39	0.000000	14.26	0.019138	4.39	0.000000
	Beaumont	73.55±12.65	35.07∼95.29	72.66						
1000 Seed weight (g)	Stuttgart	25.77±5.07	11.17∼44.74	91.79	69.00	0.000000	75.18	0.000477	3.94	0.000000
	Beaumont	24.41±4.66	12.32∼43.86	95.52						
Kernels/cm panicle	Stuttgart	7.30±1.80	3.25∼14.61	84.71	28.72	0.000000	218.17	0.000104	3.60	0.000000
	Beaumont	6.31±1.63	2.80∼12.27	87.02						
Kernels/branch panicle	Stuttgart	17.88±4.24	11.56∼37.10	82.66	19.90	0.000000	353.27	0.000058	4.31	0.000000
	Beaumont	14.67±2.98	9.61∼23.23	77.42						
Grain weight/panicle (g)	Stuttgart	3.79±1.18	0.68∼8.62	82.29	21.86	0.000000	241.69	0.000075	3.94	0.000000
	Beaumont	2.75±0.95	0.63∼6.27	80.72						

Six traits were significantly correlated with harvest index and these correlation directions were the same across the two locations. The correlations with harvest index were negative for heading (−0.46 at Stuttgart and −0.61 at Beaumont), plant height (−0.50 and −0.50), plant weight (−0.36 and −0.30), panicle length (−0.45 and −0.32), while positive for seed set (0.52 and 0.61) and grain weight/panicle (0.32 and 0.40) (Figure 3A, B). In the PCA based on phenotypic traits of 203 mini-core accessions, four traits negatively correlated with harvest index were plotted on opposing axis from harvest index (Figure 3A,B). Conversely, two traits positively correlated with harvest index were plotted in the same axis relatively close to harvest index.

### Model comparison and marker-trait associations

Dimension determination for PCA indicated that different dimensions should be included for testing associations for these traits. Further, relative performance of the association mapping models was also evaluated based on the criterion BIC (Table S2). The smaller BIC indicated the better model fit [25]. Among all possible models (naive, kinship, PCA, Q, PCA+kinship and Q+kinship), naive and kinship models showed the highest BIC value. The four other models (PCA, Q, P+kinship and Q+kinship) had a better performance, indicated by smaller BIC values. The model installed with kinship had a slightly higher BIC than the one without kinship. The PCA models containing different dimensions for different traits had the lowest BIC value. Thus, the PCA model was selected to conduct association mapping for harvest index traits.

At Stuttgart, a total of 36 markers were identified to be significantly associated with harvest index traits at the 6.45×10^−3^ level of probability (the Bonferroni corrected significance level) (Table S3). Among 36 markers, seven were associated with harvest index *per se*, five with heading, three with plant height, six with plant weight, five with panicle length, nine with seed set and one with grain weight/panicle. Eight of these trait-marker associations have been reported previously (Table S3). Additionally, seven markers were consistently associated with two or more harvest index traits [39]. Out of the seven consistent markers, RM600, RM5 and RM302 were co-associated with harvest index and seed set, RM431 with heading and seed set, RM341 with plant height and panicle length, RM471 with heading and plant weight, and RM510 with three traits, plant height, harvest index and seed set.

At Beaumont, we identified 28 markers significantly associated with harvest index traits (Table S3). Among these, two were associated with harvest index, three with heading, nine with plant height, six with plant weight, four with panicle length, three with seed set and one with grain weight/panicle. At Stuttgart, eight of the trait-marker associations have been identified in previous QTL studies. Two consistent markers were RM208 co-associated with harvest index and seed set, and RM55 co-associated with plant height and plant weight.

Across two locations, the associations of RM431 with plant height, Rid12 and RM471 with plant weight, and RM24011 with panicle length were consistently true. The four markers that associated with the same trait across both locations are called “constitutive QTL” markers, while others that associated with a certain trait only at one location are called “adaptive QTL” markers [23].

### Allelic effects

**Figure 4:**
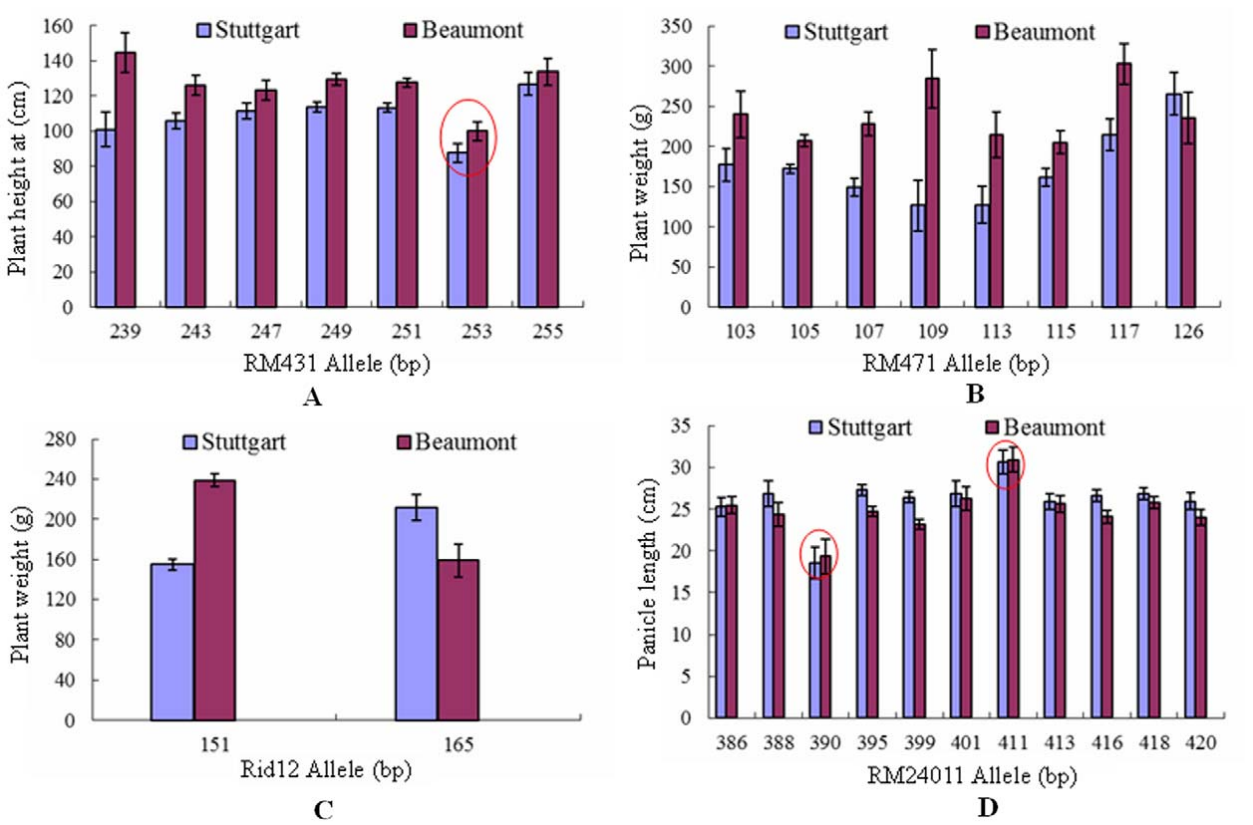
Comparisons of allelic effects of four constitutive marker loci. A: RM431 associated with plant height, B: RM471 and C: Rid12 associated with plant weight, D: RM24011 associated with panicle length constitutively at both Stuttgart, Arkansas and Beaumont, Texas.

The allelic effects of the constitutive markers associated with their traits were estimated using the least square mean (LSMEAN) of phenotypic values and are presented in Figure 4 and Table S4. For RM431, allele 253 bp had a significantly larger effect than all other 6 alleles at Beaumont and than 4 others at Stuttgart to reduce plant height. For RM24011, allele 390 bp had the greatest effect on decreasing panicle length while allele 411 bp had the largest effect on increasing panicle length at both locations. However, for Rid12, the allelic effects were opposite between two locations. Allele 151 bp of Rid12 had a decreasing effect on plant weight at Stuttgart, but an increasing effect at Beaumont instead. The 165 allele of Rid12 had an opposite effect to 151 bp on plant weight. For RM471, the allelic effects on plant weight were not consistent from one location to another. The 109 bp allele was associated with one of the lowest means for plant weight at Stuttgart, but one of the largest means for plant weight at Beaumont.

## Discussion

### Genetic diversity and genetic structure

The average number of alleles per locus was 12.86 among 203 accessions in the URMC genotyped with 155 markers. The allele number per locus is the highest among the rice collections that have been reported to date [37], [40], including an Indian germplasm collection [41], an Indonesian landrace collection [42] and a Brazilian rice core collection [43], with an exception of an Indonesian traditional and improved rice collection with 13 alleles per locus reported by Thomson et al. [44]. The average polymorphic information content (PIC) value in this study was 0.71, which is also the highest among previous studies for rice populations [37], [40], [41], [42], [44], [45] with an exception of 0.75 PIC value in a study reported by Borba et al. [43]. The wide range of genetic diversity along with the manageable number of accessions in the URMC makes it one of the best collections for mining valuable genes in rice.

Population structure is an important component in association mapping analyses because it can be a source of Type I error in an autogamous species such as barley and rice [46], [47], [48]. In this study, the 203 *O. sativa* accessions in the URMC were divided into five model-based groups from ancestry analysis (Figure 1A). Both the dendrogram tree (Figure 1B) and the PCA analysis (Figure 1C) reached similar conclusions regarding population structure in this collection. The results obtained from these three separate analyses supported each other. The classification agreed with the previous study [33] except for the group of wild relatives of rice having a high rate of rare alleles. The high rate of rare alleles was suggested by its high percentage of private alleles and the small size of the group [33]. The wild rice accessions were not integrated into association mapping since low frequency alleles are known to inflate variance estimates of linkage disequilibrium and produce a greater chance of Type I error [46], [47], [49]. In addition, the population structure was observed to be tied with geographic origins, e.g. TEJ mainly distributed in the temperate zone (Figure 2) and wild rice relatives were from a relatively isolated area (data not shown). The distinctive geographic origins corresponding to the difference of ecological environments could be partially responsible for the genetic differentiation, which in turn contributes to the different responses to environmental factors and rare alleles in the germplasm accessions of wild relative species.

### Morphological environment-sensitivity and trait-trait correlation

All 14 traits were significantly affected by environment and environment X genotype interaction, which suggested genotypic sensitivities to differences in environmental conditions at the two locations (Table 1). The sensitivity of panicle heading to temperature change and the variation of harvest index in response to photoperiod were previously observed in rice [50]. Others have reported that rice accessions derived from different geographic regions react to environmental signals differently as well [51], [52]. Information on germplasm and environmental interaction is helpful for parental selection for a specific or broad adaptation to environments.

The correlations among the 14 traits exhibited a complex relationship between pairs of traits. At both locations, the harvest index increased with an increase of seed set and grain weight/panicle, while decreased with an increase of heading, panicle length, plant height and plant weight. The negative and significant correlation between heading and harvest index was also reported in spring wheat [53], rice [54] and sorghum [11]. These studies concluded that harvest index could be easily influenced not only during the grain filling period [55], [56], but also during the period from panicle initiation to heading [54] as affected by planting dates and temperature during the growing season [57]. Plant height is another important agronomic trait that is directly linked to harvest index [9], [58]. Yoshida et al. [15] also reported a similar result to this study where harvest index was inversely correlated with plant height, which may be due to lodging in the tall varieties [54], or greater translocation of photosynthate from the vegetative tissues to grain in semi-dwarf varieties [59]]. The positive correlation between harvest index and grain weight/panicle was also reported by Sabouri et al. [10]. However, panicle length was not found to be correlated with harvest index in Marri's study [9]. Similarly, plant weight was not correlated with harvest index in Sabouri's study [10]. These different results are understandable since different materials were used in those studies. In practice, highly correlated traits, such as heading, can be used to obtain indirect estimates of harvest index when direct estimates are difficult or impractical to obtain. Thus improvement of harvest index can be manipulated indirectly. In theory, the correlation of harvest index with its related traits determined in this study, indicates an interrelationship of physiological pathways controlling these traits.

### Model comparison for association mapping of harvest index's traits

For harvest index traits, the number of dimensions in PCA was tested for each trait, and the appropriate number of dimensions was determined on the basis of BIC. Our simulated experiments showed that the dimension of PCA can exhibit phenotypic specificity. As an example with heading, the PCA model required a higher dimension number to capture the true population structure effects. Traditionally, the number of dimensions has been generally determined on the basis of random marker information without considering phenotypic information. However, the effects of population structure on different complex traits vary dramatically [60], [61] and it is logical to hypothesize that the numbers of dimensions required for cofactors in detecting marker–trait association are not necessarily the same [62].

Comparing with other five models (naive, kinship, PCA+Kinship, Q and Q+Kinship model), the PCA showed the best fit with the smallest BIC value for harvest index traits. Interestingly, correction of the kinship model was not observed to be better than the naive model. Similarly, the models with Q+kinship or PCA+kinship did not perform better than the ones with only Q or PCA, either. Shao et al. [63] also found that Q+kinship model performed similarly to the Q model alone in a rice panel. The result did not agree with some other studies on cross-pollinated plants and humans [25], [62], where the relatedness among accessions in a population is quite complex because of the mating style. The low complex relatedness in the URMC rice collection could be attributable to the restricted gene flow among these self-pollinated accessions and the diverse global origination of these accessions. Moreover, the low complex relatedness may be a result of the M strategy based on 26 phenotypic traits and 70 molecular markers [48] being used to develop this collection. This strategy is a powerful approach for selection of accessions with the most diverse alleles because it eliminates redundancies resulting from noninformative alleles that arise from co-ancestry [64]. The low-complexity relatedness was also confirmed by few secondary branches in the UPMGA tree (Figure 1B). In summary, different populations may have their own best fit model for a specific trait, which makes it necessary to compare different models.

### Genetic dissection of harvest index

Harvest index is an integrative trait including the net effect of all physiological processes during the crop cycle and its phenotypic expression is generally affected by genes responsible for non-target traits, such as heading [20], [65], plant height [20] and panicle architecture [66]. The magnitude and direction of these gene functions on different phenotypes would bear heavily on the utility of such genes for improvement of these traits. In the current study, the traits like heading, plant height, plant weight and panicle length had a strong negative correlation with harvest index, while seed set and grain weight/panicle were positively correlated with harvest index. These phenotypic correlations were consistently reflected in the identification of molecular markers associated with harvest index and related traits. For example, four consistent markers at Stuttgart, RM600, RM302, RM25, and RM431, were associated with not only harvest index itself, but also for one or more traits consistently correlated with harvest index. Another consistent marker, Rid12, associated with both heading and plant weight, was close to a reported QTL “*qHID7-1*” responsible for harvest index [67] and the gene “*Ghd7*” having major effects on grains per panicle, plant height and heading in rice [68]. At Beaumont, the consistent marker RM55, associated with both plant height and plant weight, was adjacent to a QTL “*qHID3-2*” for control of harvest index [67]. RM431 co-associated with plant height and harvest index in this study has been reported to be closely linked to gene “*sd1*” [69], [70]. The *sd1* that is involved in gibberellic acid biosynthesis decreases plant height, thus increases harvest index. The decreased height reduces lodging susceptiblity, is tolerant to heavy applications of nitrogen fertilizer, and can be planted at relatively high density, all contributing to improved grain yield that has resulted in the Green Revolution in cereal crops including rice [71].

Other markers were associated with the traits correlated with harvest index, but not with harvest index directly in this study. These markers have been reported either nearby or flanking the QTLs for harvest index. RM5, which was associated with plant height in the Stuttgart study, was close to a reported QTL for harvest index on Chr 1 [9]. RM471 associated with plant weight was close to the reported *qHID4-1* and *qHID4-2* for harvest index [67]. Furthermore, RM257 and RM22559 associated with seed set were co-localized with a known QTL on Chr 9 [9], and with *qHID8-1* [67] for harvest index, respectively. Similarly, at Beaumont, RM44 associated with plant height was close to *qHID8-1* [67], and RM263 associated with heading was adjacent to *hi2.1* [9]. The chromosomal regions where numerous correlated traits are mapped indicate either pleiotropy of a single gene or tight linkage of multiple genes. Fine-mapping of such chromosomal regions would help discern the actual genetic control of these congruent traits. Development of markers for such traits in specific regions could lead to a highly effective strategy of marker-assisted selection for improving harvest index.

### Environmental sensitivity and marker-assisted selection

Quantitative traits show a range of sensitivities to environmental changes [67]. In this study, 32 marker-trait associations were identified specifically adaptive to Stuttgart, whereas 24 marker-trait associations were adaptive to Beaumont. More importantly, we identified four constitutive markers associated with harvest index traits in both environments.

Environment-specific QTLs can be used for marker-assisted selection (MAS) at specific environments. For example, RM431 could be used to improve harvest index directly and indirectly through decreasing plant height and increasing seed set in Arkansas because it was co-associated with harvest index, plant height, and seed set. However, the constitutive marker-trait associations over multiple environments can be applied to MAS programs in a wide area. For example, results suggest that the constitutive markers Rid12 and RM471 could be used to improve harvest index indirectly through decreasing plant weight in the southern states of the USA.

Comparison of allelic effects of these constitutive markers can classify the alleles within a marker locus into superior or inferior ones, which helps decide which to use for MAS in the southern states. For example, allele 253 bp of RM431 and allele 390 bp of RM24011 had the largest effect on decreasing two traits, plant height and panicle length, negatively associated with harvest index. Thus, these superior alleles can be introduced for improvement of harvest index indirectly through decreasing the negative traits at both locations. Conversely, the allele 411 bp of RM24011 had the largest effect on increasing the panicle length and thus would not be useful for improving harvest index using MAS at either location. Interestingly, the two alleles of Rid12 associated with plant weight had opposite effects at the two locations. Allelic choice for this marker should be dependent on the particular environment targeted for breeding.

Results of the present study demonstrated that genome-wide association mapping in the URMC could complement and enrich the information derived from linkage-based QTL studies. After validation or fine mapping of these putative genomic regions, the information will help secure food production through either direct improvement of harvest index or indirect improvement via changes in seed set, grain weight per panicle, heading, plant height and weight, and panicle length using the MAS.

## Materials and Methods

### Rice association panel

Of 217 accessions in the URMC, 203 belong to O. *sativa* whereas the remaining belongs to other species in *Oryza*. Pure seed of these accessions were provided by the Genetic Stock *Oryza* Collection (GSOR) (www.ars.usda.gov/spa/dbnrrc/gsor) with cultivar name or designation, accession number, registration year, place of origin, longitude and latitude of origin, pedigree or genetic background (if available), morphological characteristics and references. The GSOR supplies seeds for research purposes to national and international users upon to request. In this study, only 203 *O. sativa* accessions were used for the following analysis because the wild relatives, *O. glaberrima*, *nivara*, *rufipogon*, *glumaepatula* and *latifolia*, contain many rare alleles. Rare alleles are one of the factors that increase the risk of Type I errors or spurious associations [47].

### Location and field experiment

Evaluations were conducted for 14 traits in two field locations, USDA-ARS Dale Bumpers National Rice Research Center near Stuttgart, Arkansas and USDA-ARS Rice Research Unit near Beaumont, Texas during the 2009 growing season. The Stuttgart test site is located at N 34°27′44″ and W 91°24′59″, representing a temperate climate with a 243 d frost free period and average temperature of 23.9 C during the growing season. The Beaumont test site is located at N 30°03′47″ and W 94°17′45″, representing a subtropical climate with a 253 d frost free period and an average temperature of 26.1 C during the growing season. The experiments at both locations utilized a randomized complete block design having three replications with nine plants spaced 0.3×0.6 m in each plot. Three seeds were sown in each of nine hills in a plot using a Hege 1000 grain drill planter on April 23 and May 6 of 2009 at Stuttgart and Beaumont, respectively. Each hill was thinned to a single plant right after the permanent flood was applied at five leaf stage. Before flooding, fertilizer at 55 kg ha^−1^ of nitrogen as urea was applied. Weeds were controlled at both pre-planting and pre-flooding stages with locally recommended herbicides.

### Phenotyping

Data collection followed procedures described by Yan et al. [72], [73] with modifications. Heading was recorded as the number of days when 50% of the panicles in a plot had begun to emerge from the boot. Meanwhile, three plants were selected from the 9 in each plot and their main panicles were marked. Each plant was then bagged at the top to avoid panicle damage and supported by a bamboo pole to avoid lodging. Each plant was manually cut at ground level when mature and air-dried for two months before recording plant weight (g). Then, plant height (cm) was measured from the base to the panicle tip, the main panicle was removed at the panicle node and tillers of the plant were recorded before being threshed. Grain yield (g) was measured as total weight after the threshed grains were cleaned by an Almaco seed cleaner, plus seed weight of the removed main panicle. Harvest index (%) was calculated as the ratio of grain yield to plant weight. Each main panicle was measured for its length (cm), counted for its primary and secondary branches and manually threshed for kernels. All kernels from the panicle were placed in a cup half full of water and the cup was stirred with a spoon. Blank kernels floated to the top of the water and filled kernels sank to the bottom. The number of each was recorded after they were dried at 50°C for 12 hrs. Seed weight (mg) was determined by the filled kernel weight divided by its number, and seed set (%) was expressed by a ratio of the filled kernels to the total kernels including both filled and unfilled in the panicle. Panicle length and branch data were used to generate kernels/cm panicle and kernels/branch panicle using the total kernels.

### Genotyping

Bulk tissue from five plants was collected from each accession as described by Brondani et al. [74] and total genomic DNA was extracted using a rapid alkali extraction procedure [75]. The bulked DNA allowed identification of the origin of heterogeneity, which can result from the presence of heterozygous individuals or from a mix of individuals with different homozygous alleles [76]. The 155 molecular markers covering the entire rice genome, approximately one marker per 10 cM on average, were used to genotype 203 accessions in the URMC. Among the markers, 149 SSRs were obtained from the Gramene database (http://www.gramene.org/), and five SSRs (AP5652-1, AP5652-2, AL606682-1, con673 and LJSSR1) were amplified in house [33]. The remaining was an *indel* at the *Rc* locus, named *Rid 12* and is responsible for rice pericarp color. Polymerase chain reaction (PCR) marker amplifications were performed as described in Agrama et al. [34]. The genetic positions and physical positions of these markers were estimated using the map of Cornell SSR 2001 and the map of Gramene Annotated Nipponbare Sequence 2009, respectively (http://www.gramene.org/). Markers labeled with different colored fluorescence and that amplified products with size differences of 20 bp or more were multiplexed together post PCR.

### Statistical analysis

#### Marker and phenotype profile

Genetic distance was calculated from the 155 molecular markers using Nei distance [36]. Phylogenetic reconstruction was based on the UPGMA method implemented in *PowerMarker* version 3.25 [77]. *PowerMarker* was also used to calculate the average number of alleles, gene diversity, and polymorphism information content (PIC) values. The tree to visualize the phylogenetic distribution of accessions and ancestry groups was constructed using MEGA version 4 [78].

Each of the 14 phenotypic traits was modeled independently with the MIXED procedure in SASv.9.2, where genotype, location and interaction of location with genotype were defined as fixed effects while replication within a location (block effect) was a random effect. Broad-sense heritability was calculated using formula H^2^ = σ_g_^2^/(σ_g_^2^+σ_e_^2^/n), where σ_g_^2^ as the genotypic variance, σ_e_^2^ as the environmental variance and n as the number of replications [79]. Spearman rank correlation coefficients between each pair of the 14 traits were calculated using the mean of 9 plants, 3 in each of three replications for an accession, using the CORR procedure in SASv.9.2. Correlation coefficients for the traits that significantly correlated with harvest index were displayed graphically using principal components analysis (PCA) performed with *NTSYSpc* software version 2.11 [80].

#### Population structure

The model-based program *STRUCTURE* [81] was used to infer population structure using a burn-in of 100,000, a run length of 100,000, and a model allowing for admixture and correlated allele frequencies. The number of groups (K) was set from 1 to 10, with ten independent runs each. The most probable structure number of (K) was calculated based on Evanno et al. [82] using an ad hoc statistic *D(K)*, assisted with *L(K)*, *L′(K)* and *(L″K)*. The *D(K)* perceives the rate of change in log probability of the data between successive (K) values rather than just the log probability of the data. Determination of mixed ancestry (an accession unable to be clearly assigned to only one group) was based on 60% (Q) as a threshold to consider an individual with its inferred ancestry from one single group. Principal component analysis (PCA), that summarizes the major patterns of variation in a multi-locus data set, was performed with *NTSYSpc* software version 2.11 [80]. The first three principal components were used to visualize the dispersion of the mini core accessions in a graph. Each accession was assigned into a group according to its maximum ancestry index assessed by *STRUCTURE* for the following linkage disequilibrium analysis.

#### Model comparison and association mapping

Following the procedures previously recommended [25], [62] for various mixed models, we tested a subpopulation membership percentage (Q), PCA as fixed covariates and kinship (K) as a random effect. The kinship was calculated using SPAGeDi [83]. Phenotypic data were also incorporated into the process to determine the final number of dimensions for PCA based on Bayesian information criterion (BIC) [62]. The best fit model for each trait was determined based on the BIC among six models, naive, Kinship, PCA, PCA+Kinship, Q and Q+Kinship [25], [84]. The selected model was then used to map the SSR markers significantly associated with harvest index's traits. The association analysis was conducted using the MIXED procedure in SASv.9.2. For multiple testing, *P* values were compared to the Bonferroni threshold (1/155 = 6.45×10^−3^) to identify statistically significant loci. Allelic effects at marker loci were compared using the LSMEANS and pdiff option in the MIXED procedure, using Saxton's PDMIX800 SAS macro [85].

## Supporting Information

Table S1 A. Spearman correlation for each pair of 14 traits evaluated at Stuttgart, Arkansas in 2009. B. Spearman correlation for each pair of 14 traits evaluated at Beaumont, Texas in 2009.
(DOC)

Table S2 **Fitness analysis of mapping model for harvest index traits using Bayesian information criterion (BIC) in both Arkansas and Texas.**
(DOC)

Table S3 **The marker loci associated with harvest index traits at Stuttgart, Arkansas and Beaumont, Texas in 2009.**
(DOC)

Table S4 **Comparison of allelic effect of four constitutive marker loci at two locations, Stuttgart, Arkansas and Beaumont, Texas.** RM431 for Plant height, RM471 and Rid12 for Plant weight, and RM24011 for Panicle length.
(DOC)
