# Supplementary material for: Unraveling the Complex Trait of Harvest Index with Association Mapping in Rice (Oryza sativa L.)
Source: PLoS One. 2012 Jan 23;7(1):e29350. doi: 10.1371/journal.pone.0029350 (PMC3264563; doi:10.1371/journal.pone.0029350)
Supplement: Table S1 — A. Spearman correlation for each pair of 14 traits evaluated at Stuttgart, Arkansas in 2009. B. Spearman correlation for each pair of 14 traits evaluated at Beaumont, Texas in 2009. (DOC) [file pone.0029350.s001.doc]

| Supplemental Table 1A. Spearman correlation for each pair of 14 traits among 203 accessions in the USDA rice mini-core collection evaluated in replicated tests at Stuttgart, Arkansas in 2009. | | | | | | | | | | | | | | |
| --- | --- | --- | --- | --- | --- | --- | --- | --- | --- | --- | --- | --- | --- | --- |
| Trait | Heading | Plant height | Plant weight | Tillers | Grain yield | Harvest index | Panicle length | Panicle branches | Kernels/ panicle | Seed set | 1000 Seed weight | Kernels/cm panicle | Kernels/branch panicle | Grain weight/panicle |
| Heading |  | 0.3188 | 0.3011 | -0.0874 | -0.0267 | -0.462 | 0.5703 | 0.4616 | 0.4134 | -0.3554 | -0.0237 | 0.1573 | 0.1385 | 0.1143 |
| Plant height | <.0001 |  | 0.6649 | 0.344 | 0.4319 | -0.4967 | 0.6988 | 0.1807 | 0.3501 | -0.1432 | -0.1533 | 0.0137 | 0.2652 | 0.1529 |
| Plant weight | <.0001 | <.0001 |  | 0.8006 | 0.8122 | -0.3603 | 0.5407 | -0.0511 | 0.3667 | -0.2377 | -0.2998 | 0.1116 | 0.4427 | 0.0076 |
| Tillers | 0.2257 | <.0001 | <.0001 |  | 0.7663 | -0.1275 | 0.1674 | -0.4102 | 0.0421 | -0.1678 | -0.4096 | -0.0653 | 0.3444 | -0.3303 |
| Grain yield | 0.7146 | <.0001 | <.0001 | <.0001 |  | 0.1316 | 0.296 | -0.1682 | 0.2434 | 0.0834 | -0.1511 | 0.1226 | 0.4042 | 0.2381 |
| Harvest index | <.0001 | <.0001 | <.0001 | 0.0796 | 0.0703 |  | -0.447 | -0.0929 | -0.1736 | 0.5175 | 0.1828 | 0.0805 | -0.1136 | 0.3195 |
| Panicle length | <.0001 | <.0001 | <.0001 | 0.021 | <.0001 | <.0001 |  | 0.2579 | 0.5187 | -0.2894 | -0.0438 | 0.0518 | 0.392 | 0.2676 |
| Panicle branches | <.0001 | 0.0126 | 0.4836 | <.0001 | 0.0203 | 0.2022 | 0.0003 |  | 0.4946 | -0.1373 | 0.0715 | 0.4585 | -0.1432 | 0.427 |
| Kernels/ panicle | <.0001 | <.0001 | <.0001 | 0.5638 | 0.0007 | 0.0166 | <.0001 | <.0001 |  | -0.3166 | -0.3785 | 0.8499 | 0.7409 | 0.4352 |
| Seed set | <.0001 | 0.0487 | 0.001 | 0.0207 | 0.2527 | <.0001 | <.0001 | 0.0589 | <.0001 |  | 0.1402 | -0.171 | -0.2603 | 0.353 |
| 1000 Seed weight | 0.7456 | 0.0347 | <.0001 | <.0001 | 0.0374 | 0.0116 | 0.5481 | 0.3269 | <.0001 | 0.0537 |  | -0.4089 | -0.4586 | 0.4002 |
| Kernels/cm panicle | 0.0302 | 0.8515 | 0.1252 | 0.3705 | 0.0921 | 0.2697 | 0.4781 | <.0001 | <.0001 | 0.0183 | <.0001 |  | 0.6192 | 0.3837 |
| Kernels/branch panicle | 0.0567 | 0.0002 | <.0001 | <.0001 | <.0001 | 0.1186 | <.0001 | 0.0487 | <.0001 | 0.0003 | <.0001 | <.0001 |  | 0.1818 |
| Grain weight/panicle | 0.1165 | 0.0352 | 0.9171 | <.0001 | 0.0009 | <.0001 | 0.0002 | <.0001 | <.0001 | <.0001 | <.0001 | <.0001 | 0.0121 |  |
| Above diagonal: Spearman correlation coefficient (r) | | | | | | | | | | | | | | |
| Below diagonal: Corresponding p-value for significant test | | | | | | | | | | | | | | |

| Supplemental Table 1B. Spearman correlation for each pair of 14 traits among 203 accessions in the USDA rice mini-core collection evaluated in replicated tests at Beaumont, Texas in 2009. | | | | | | | | | | | | | | |
| --- | --- | --- | --- | --- | --- | --- | --- | --- | --- | --- | --- | --- | --- | --- |
| Trait | Heading | Plant height | Plant weight | Tillers | Grain yield | Harvest index | Panicle length | Panicle branches | Kernels/panicle | Seed set | 1000 Seed weight | Kernels/cm panicle | Kernels/branch panicle | Seed weight/ Panicle |
| Heading |  | 0.4839 | 0.5726 | 0.1604 | -0.0164 | -0.6112 | 0.4901 | 0.3518 | 0.2618 | -0.3727 | -0.0873 | 0.0811 | 0.0656 | -0.0286 |
| Plant height | <.0001 |  | 0.5925 | 0.0964 | 0.2118 | -0.5045 | 0.6439 | 0.3898 | 0.3371 | -0.2239 | 0.0109 | 0.0981 | 0.1339 | 0.1408 |
| Plant weight | <.0001 | <.0001 |  | 0.6261 | 0.6431 | -0.2962 | 0.4328 | 0.2812 | 0.2467 | -0.0964 | -0.1951 | 0.1102 | 0.1104 | 0.0666 |
| Tillers | 0.0319 | 0.1966 | <.0001 |  | 0.5509 | 0.0138 | -0.0151 | -0.2978 | -0.2667 | 0.1626 | -0.5524 | -0.2736 | -0.0938 | -0.424 |
| Grain yield | 0.8294 | 0.0049 | <.0001 | <.0001 |  | 0.3813 | 0.2121 | 0.204 | 0.2732 | 0.3839 | -0.2029 | 0.2318 | 0.2153 | 0.3488 |
| Harvest index | <.0001 | <.0001 | <.0001 | 0.8582 | <.0001 |  | -0.322 | -0.082 | 0.0731 | 0.6138 | -0.0013 | 0.2366 | 0.1715 | 0.3956 |
| Panicle length | <.0001 | <.0001 | <.0001 | 0.8433 | 0.005 | <.0001 |  | 0.3494 | 0.4235 | -0.2328 | 0.0644 | 0.0131 | 0.2924 | 0.2436 |
| Panicle branches | <.0001 | <.0001 | 0.0002 | <.0001 | 0.0069 | 0.2863 | <.0001 |  | 0.668 | -0.13 | 0.1779 | 0.5835 | 0.053 | 0.6227 |
| Kernels/panicle | 0.0004 | <.0001 | 0.0011 | 0.0004 | 0.0003 | 0.3266 | <.0001 | <.0001 |  | -0.0876 | -0.1267 | 0.8827 | 0.7369 | 0.6769 |
| Seed set | <.0001 | 0.0032 | 0.2097 | 0.0336 | <.0001 | <.0001 | 0.0021 | 0.0892 | 0.2383 |  | -0.1776 | 0.0168 | -0.021 | 0.3305 |
| 1000 Seed weight | 0.2413 | 0.8877 | 0.0106 | <.0001 | 0.008 | 0.9862 | 0.4016 | 0.0196 | 0.0875 | 0.0162 |  | -0.1737 | -0.3096 | 0.3647 |
| Kernels/cm panicle | 0.2766 | 0.2019 | 0.1514 | 0.0003 | 0.0024 | 0.0013 | 0.8649 | <.0001 | <.0001 | 0.821 | 0.0187 |  | 0.6711 | 0.6345 |
| Kernels/branch panicle | 0.3787 | 0.0807 | 0.1504 | 0.2225 | 0.0048 | 0.0206 | <.0001 | 0.4898 | <.0001 | 0.778 | <.0001 | <.0001 |  | 0.3727 |
| Seed weight/ Panicle | 0.7015 | 0.0663 | 0.3868 | <.0001 | <.0001 | <.0001 | 0.0013 | <.0001 | <.0001 | <.0001 | <.0001 | <.0001 | <.0001 |  |
| Above diagonal: Spearman correlation coefficient (r) | | | | | | | | | | | | | | |
| Below diagonal: Corresponding p-value for significant test | | | | | | | | | | | | | | |
