# Supplementary material for: Unraveling the Complex Trait of Harvest Index with Association Mapping in Rice (Oryza sativa L.)
Source: PLoS One. 2012 Jan 23;7(1):e29350. doi: 10.1371/journal.pone.0029350 (PMC3264563; doi:10.1371/journal.pone.0029350)
Supplement: Table S2 — Fitness analysis of mapping model for harvest index traits using Bayesian information criterion (BIC) in both Arkansas and Texas. (DOC) [file pone.0029350.s002.doc]

| Supplemental Table 2. Fitness analysis of mapping model for harvest index’s traits using Bayesian information criterion (BIC) among 203 accessions genotyped with 155 molecular markers and phenotyped in both Arkansas and Texas | | | | | | | | |
| --- | --- | --- | --- | --- | --- | --- | --- | --- |
| Location | Trait Model | Heading | Plant height | Plant weight | Harvest index | Panicle length | Seed set | Grain weight/panicle |
| Stuttgart | Naïve | 1655.56 | 1630.18 | 2234.42 | -429.67 | 1006.92 | 1487.89 | 587.16 |
|  | Kinship | 1660.78 | 1635.40 | 2239.69 | -424.46 | 1012.12 | 1493.10 | 592.37 |
|  | Q | 1561.54 | 1562.70 | 2091.12 | -437.91 | 939.28 | 1422.41 | 530.47 |
|  | Q+Kinship | 1566.76 | 1567.92 | 2096.40 | -432.71 | 944.49 | 1427.61 | 535.67 |
|  | PCA | 1502.19 | 1498.72 | 2017.11 | -455.06 | 910.73 | 1385.01 | 535.41 |
|  | PCA+Kinship | 1507.42 | 1503.94 | 2022.39 | -449.86 | 915.94 | 1390.21 | 540.62 |
|  |  |  |  |  |  |  |  |  |
| Beaumont | Naïve | 1723.95 | 1567.75 | 2014.70 | -270.19 | 900.54 | 1367.68 | 492.38 |
|  | Kinship | 1729.20 | 1572.90 | 2019.86 | -265.03 | 905.67 | 1372.84 | 497.54 |
|  | Q | 1620.35 | 1491.88 | 1885.09 | -275.10 | 848.71 | 1308.60 | 433.11 |
|  | Q+Kinship | 1625.60 | 1497.04 | 1890.25 | -269.94 | 853.84 | 1313.76 | 438.28 |
|  | PCA | 1552.14 | 1424.66 | 1832.52 | -283.86 | 815.62 | 1281.08 | 431.00 |
|  | PCA+Kinship | 1557.39 | 1429.82 | 1837.67 | -278.70 | 820.74 | 1286.25 | 436.17 |
|  | | | | | | | | |
