# Supplementary material for: Unraveling the Complex Trait of Harvest Index with Association Mapping in Rice (Oryza sativa L.)
Source: PLoS One. 2012 Jan 23;7(1):e29350. doi: 10.1371/journal.pone.0029350 (PMC3264563; doi:10.1371/journal.pone.0029350)
Supplement: Table S3 — The marker loci associated with harvest index traits at Stuttgart, Arkansas and Beaumont, Texas in 2009. (DOC) [file pone.0029350.s003.doc]

| Table S3. The marker loci associated with harvest index traits at Stuttgart, Arkansas and Beaumont, Texas in 2009. | | | | | | | | | | | | | |
| --- | --- | --- | --- | --- | --- | --- | --- | --- | --- | --- | --- | --- | --- |
| Trait | Marker | Stuttgart, Arkansas | | | | | Trait | Marker | Beaumont, Texas | | | | |
|  |  | Chr | cM | ProbF |  | Annotation |  |  | Chr | cM | ProbF |  | Annotation |
| Heading | RM3562 | 3 | 52.0 | 0.0001 | # |  | Heading | RM178 | 5 | 104.4 | 0.0026 |  |  |
|  | Rid12 | 7 | 41.0 | 0.0009 | # |  |  | RM263 | 2 | 105.0 | 0.0029 |  | *qHD-2* [86] |
|  | RM3726 | 12 | 91.0 | 0.0018 |  |  |  | RM279 | 2 | 14.0 | 0.0044 |  |  |
|  | RM162 | 6 | 88.0 | 0.0034 |  |  |  |  |  |  |  |  |  |
|  | RM125 | 7 | 41.0 | 0.0040 |  | *dth7.1* [87] |  |  |  |  |  |  |  |
|  |  |  |  |  |  |  |  |  |  |  |  |  |  |
| Plant height | RM431 | 1 | 154.6 | 0.0001 | #* | *qPHT-1* [87], *ph1.2* [88], *sd1*[69] | Plant height | RM44 | 8 | 53.0 | 0.0002 |  | *ph-8*[89] |
|  | RM509 | 5 | 59.0 | 0.0043 | # |  |  | RM431 | 1 | 154.6 | 0.0003 | * | *qPHT-1*[88], *ph1.2* [87], *sd1*[69] |
|  | RM5 | 1 | 98.5 | 0.0061 |  | *ph1.1*[87] |  | RM145 | 2 | 36.8 | 0.0008 |  |  |
|  |  |  |  |  |  |  |  | RM55 | 3 | 129.0 | 0.0019 | # | *qPHT3-1*, *qPHT3-2*[65] |
|  |  |  |  |  |  |  |  | RM316 | 9 | 0.0 | 0.0020 |  | Mei et al.[90] |
|  |  |  |  |  |  |  |  | RM3739 | 12 | 97.0 | 0.0035 |  |  |
|  |  |  |  |  |  |  |  | RM3726 | 12 | 91.0 | 0.0045 |  |  |
|  |  |  |  |  |  |  |  | RM284 | 8 | 78.5 | 0.0047 |  | *ph8.1*[91] [92] |
|  |  |  |  |  |  |  |  | RM1339 | 1 | 147.5 | 0.0056 |  |  |
|  |  |  |  |  |  |  |  |  |  |  |  |  |  |
| Plant weight | RM7003 | 12 | 41.0 | 0.0000 |  |  | Plant weight | Rid12 | 7 | 41.0 | 0.0001 | * |  |
|  | Rid12 | 7 | 41.0 | 0.0006 | #* |  |  | RM452 | 2 | 44.0 | 0.0011 |  |  |
|  | RM471 | 4 | 45.0 | 0.0010 | * |  |  | RM55 | 3 | 129.0 | 0.0029 | # | [92] |
|  | RM279 | 2 | 14.0 | 0.0014 |  |  |  | RM105 | 9 | 41.0 | 0.0039 |  |  |
|  | RM224 | 11 | 115.0 | 0.0033 |  | [92] |  | RM3562 | 3 | 52.0 | 0.0046 |  |  |
|  | RM527 | 6 | 59.0 | 0.0048 |  |  |  | RM471 | 4 | 45.0 | 0.0048 | * |  |
|  |  |  |  |  |  |  |  |  |  |  |  |  |  |
| Harvest index | RM25 | 8 | 36.0 | 0.0001 | # |  | Harvest index | RM208 | 2 | 154.1 | 0.0009 | # |  |
|  | RM600 | 1 | 50.0 | 0.0001 | # |  |  | RM1148 | 8 | 30.0 | 0.0013 |  |  |
|  | RM3217 | 4 | 101.0 | 0.0003 |  |  |  |  |  |  |  |  |  |
|  | RM431 | 1 | 154.6 | 0.0003 | # |  |  |  |  |  |  |  |  |
|  | RM6863 | 8 | 16.4 | 0.0010 |  |  |  |  |  |  |  |  |  |
|  | RM302 | 1 | 135.8 | 0.0040 | # |  |  |  |  |  |  |  |  |
|  | RM105 | 9 | 41.0 | 0.0044 |  |  |  |  |  |  |  |  |  |
|  |  |  |  |  |  |  |  |  |  |  |  |  |  |
| Panicle length | RM510 | 6 | 11.5 | 0.0003 |  |  | Panicle length | RM247 | 12 | 26.7 | 0.0006 |  | *qPL-12* [93] |
|  | RM3739 | 12 | 97.0 | 0.0009 |  |  |  | RM24011 | 9 | 27.4 | 0.0007 | * |  |
|  | RM24011 | 9 | 27.4 | 0.0033 | * |  |  | RM162 | 6 | 88.0 | 0.0031 |  | [94] |
|  | RM509 | 5 | 59.0 | 0.0036 | # |  |  | RM283 | 1 | 25.4 | 0.0060 |  | [94] |
|  | RM245 | 9 | 91.8 | 0.0040 |  |  |  |  |  |  |  |  |  |
|  |  |  |  |  |  |  |  |  |  |  |  |  |  |
| Seed set | RM271 | 10 | 42.0 | 0.0000 |  | *pss10.1*[95] | Seed set | RM527 | 6 | 59.0 | 0.0005 |  |  |
|  | RM257 | 9 | 65.0 | 0.0001 |  | [96] |  | RM3558 | 4 | 69.8 | 0.0006 |  |  |
|  | RM302 | 1 | 135.8 | 0.0018 | # |  |  | RM208 | 2 | 154.1 | 0.0054 | # | *ss2*[97] |
|  | RM555 | 2 | 22.0 | 0.0024 |  | [98] |  |  |  |  |  |  |  |
|  | RM431 | 1 | 154.6 | 0.0028 | # |  |  |  |  |  |  |  |  |
|  | RM22559 | 8 | 42.3 | 0.0045 |  |  |  |  |  |  |  |  |  |
|  | RM25 | 8 | 36.0 | 0.0050 | # | *pss8.1*[99] |  |  |  |  |  |  |  |
|  | RM3562 | 3 | 52.0 | 0.0055 | # |  |  |  |  |  |  |  |  |
|  | RM600 | 1 | 50.0 | 0.0061 | # |  |  |  |  |  |  |  |  |
|  |  |  |  |  |  |  |  |  |  |  |  |  |  |
| Grain weight/panicle | RM341 | 2 | 70.0 | 0.0002 |  |  | Grain weight/panicle | OSR13 | 3 | 36.0 | 0.0045 |  |  |
| #: Consistent marker co-associated with two or more traits | | | | | | | | | | | | | |
| *: Constitutive marker associated with the same traits at both Stuttgart, Arkansas and Beaumont, Texas. | | | | | | | | | | | | | |
| Annotation: the markers reported either flanking or co-located to the corresponding QTL in previous reports.  65. Hemamalini GS, Shashidhar HE, Hittalmani S (2000) Molecular marker assisted tagging of morphological and physiological traits under two contrasting moisture regimes at peak vegetative stage in rice (*Oryza sativa* L.). Euphytica 112: 69-78.  69. Peng J, Richards DE, Hartley NM, Murphy GP, Devos KM, et al. (1999) 'Green revolution' genes encode mutant gibberellin response modulators. Nature 400: 256-261. | | | | | | | | | | | | | |

86. Zou JH, Pan XB, Chen ZX, Xu JY, Lu JF, et al. (2000) Mapping quantitative trait loci controlling sheath blight resistance in two rice cultivars (*Oryza sativa* L.) Theor Appl Genet 101: 569-573.

87. Moncada P, Martinez CP, Borrero J, Chatel M, Gauch H, et al. (2001) Quantitative trait loci for yield and yield components in an *Oryza sativa* X *Oryza rufipogon* BC2F2 population evaluated in an upland environment. Theor Appl Genet 102: 41-42.

88. Hittalmani S, Shashidhar HE, Bagali PG, Huang N, Sidhu JS, et al. (2002) Molecular mapping of quantitative trait loci for plant growth, yield and yield related traits across three diverse locations in a doubled haploid rice population. Euphytica 125: 207-214.

89. Lu CF, Shen LH, Tan ZB, Xu YB, He P, et al. (1997) Comparative mapping of QTLs for agronomic traits of rice across environments by using a doubled-haploid population. Theor Appl Genet 94: 145-150.

90. Mei HW, Luo LJ, Ying CS, Wang YP, Yu XQ, et al. (2003) Gene actions of QTLs affecting several agronomic traits resolved in a recombinant inbred rice population and two testcross populations. Theor Appl Genet 107: 89-101.

91. Thomson MJ, Tai TH, McClung AM, Lai XH, Hinga ME, et al. (2003) Mapping quantitative trait loci for yield, yield components and morphological traits in an advanced backcross population between *Oryza rufipogon* and the *Oryza sativa* cultivar Jefferson Theor Appl Genet 107: 479-493.

92. Lian X, Xing Y, Yan H, Xu C, Li X, et al. (2005) QTLs for low nitrogen tolerance at seedling stage identified using a recombinant inbred line population derived from an elite rice hybrid. Theor Appl Genet 112: 85-96.

93. Suh JP, Ahn SN, Cho YC, Kang KH, Choi IS, et al. (2005) Mapping of QTLs for yield traits using an advanced backcross population from a cross between *Oryza sativa* and *O. glaberrima.* Korean J Breed 37: 214-220.

94. Jiang GH, Xu CG, Li XH, He YQ (2004) Characterization of the genetic basis for yield and its component traits of rice revealed by doubled haploid population. Acta Genetica Sinica 31: 63-72.

95. Thomson MJ, Tai TH, McClung AM, Lai XH, Hinga ME, et al. (2003) Mapping quantitative trait loci for yield, yield components and morphological traits in an advanced backcross population between *Oryza rufipogon* and the *Oryza sativa* cultivar Jefferson Theor Appl Genet 107: 479-493.

96. Li JZ, Zheng XW, Zhu LH, He P, Lu RL (1999) Identification and interaction analysis of six agronomic trait loci of rice based on a recombinant inbred population. Acta Bot Sinica 41 1199-1203.

97. Xing YZ, Xu CG, Hua JP, Tan YF (2001) Analysis of QTL x environment interaction for rice panicle characteristics. Acta genetica Sinica 28: 439-446.

98. Septiningsih EM, Prasetiyono J, Lubis E, Tai TH, Tjubaryat T, et al. (2003) Identification of quantitative trait loci for yield and yield components in an advanced backcross population derived from the *Oryza sativa* variety IR64 and the wild relative *O. rufipogon*. Theor Appl Genet 107: 1419-1432.

99. Xiao J, Li J, Grandillo S, Ahn SN, Yuan L, et al. (1998) Identification of trait-improving quantitative trait loci alleles from a wild rice relative, *Oryza rufipogon*. Genetics 150: 899-909.
