# Supplementary material for: Unraveling the Complex Trait of Harvest Index with Association Mapping in Rice (Oryza sativa L.)
Source: PLoS One. 2012 Jan 23;7(1):e29350. doi: 10.1371/journal.pone.0029350 (PMC3264563; doi:10.1371/journal.pone.0029350)
Supplement: Table S4 — Comparison of allelic effect of four constitutive marker loci at two locations, Stuttgart, Arkansas and Beaumont, Texas. RM431 for Plant height, RM471 and Rid12 for Plant weight, and RM24011 for Panicle length. (DOC) [file pone.0029350.s004.doc]

| Table S4. Comparison of allelic effect of four constitutive marker loci at two locations, Stuttgart, Arkansas and Beaumont, Texas. RM431 for Plant height, RM471 and Rid12 for Plant weight, and RM24011 for Panicle length | | | | | | | | | |
| --- | --- | --- | --- | --- | --- | --- | --- | --- | --- |
| Marker | Allele (bp) | Stuttgart, Arkansas | | | Marker | Allele (bp) | Beaumont, Texas | | |
|  |  | Mean | SD |  |  |  | Mean | SD |  |
| RM431 | 239 | 100.93 | 10.05 | AB | RM431 | 239 | 144.46 | 11.35 | A |
|  | 243 | 105.69 | 4.65 | AB |  | 243 | 125.80 | 5.62 | A |
|  | 247 | 111.21 | 4.51 | A |  | 247 | 123.11 | 5.47 | A |
|  | 249 | 113.63 | 2.83 | A |  | 249 | 129.26 | 3.43 | A |
|  | 251 | 113.33 | 2.33 | A |  | 251 | 127.45 | 2.72 | A |
|  | 253 | 87.67 | 5.36 | B |  | 253 | 99.84 | 5.56 | B |
|  | 255 | 126.76 | 6.44 | A |  | 255 | 133.73 | 7.69 | A |
|  |  |  |  |  |  |  |  |  |  |
| RM471 | 103 | 177.41 | 20.39 | ABC | RM471 | 103 | 239.82 | 28.64 | AB |
|  | 105 | 172.01 | 5.84 | BC |  | 105 | 207.55 | 7.47 | B |
|  | 107 | 148.58 | 11.24 | C |  | 107 | 228.55 | 14.73 | AB |
|  | 109 | 126.38 | 31.18 | BC |  | 109 | 284.74 | 36.51 | AB |
|  | 113 | 127.08 | 22.79 | C |  | 113 | 214.46 | 28.15 | AB |
|  | 115 | 161.16 | 11.52 | BC |  | 115 | 205.07 | 14.38 | B |
|  | 117 | 214.50 | 20.06 | AB |  | 117 | 302.83 | 25.04 | A |
|  | 126 | 265.70 | 26.97 | A |  | 126 | 235.60 | 31.66 | AB |
|  |  |  |  |  |  |  |  |  |  |
| Rid12 | 151 | 154.73 | 5.24 | B | Rid12 | 151 | 238.84 | 6.75 | A |
|  | 165 | 211.88 | 13.09 | A |  | 165 | 158.72 | 16.16 | B |
|  |  |  |  |  |  |  |  |  |  |
| RM24011 | 386 | 25.28 | 1.11 | B | RM24011 | 386 | 25.49 | 0.98 | B |
|  | 388 | 26.87 | 1.50 | AB |  | 388 | 24.40 | 1.42 | BC |
|  | 390 | 18.61 | 1.90 | C |  | 390 | 19.34 | 2.10 | C |
|  | 395 | 27.28 | 0.63 | AB |  | 395 | 24.74 | 0.58 | BC |
|  | 399 | 26.43 | 0.68 | B |  | 399 | 23.18 | 0.65 | BC |
|  | 401 | 26.83 | 1.52 | AB |  | 401 | 26.26 | 1.40 | AB |
|  | 411 | 30.68 | 1.39 | A |  | 411 | 30.93 | 1.46 | A |
|  | 413 | 25.89 | 0.97 | B |  | 413 | 25.64 | 1.01 | B |
|  | 416 | 26.67 | 0.71 | AB |  | 416 | 24.10 | 0.75 | BC |
|  | 418 | 26.87 | 0.72 | AB |  | 418 | 25.77 | 0.72 | B |
|  | 420 | 25.95 | 0.97 | B |  | 420 | 24.01 | 0.98 | BC |
| LSmeans in a column followed by the same letter are not significantly different at 0.001 probability | | | | | | | | | |
